# Fractional Derivatives Application to Image Fusion Problems

**DOI:** 10.3390/s22031049

**Published:** 2022-01-28

**Authors:** Szymon Motłoch, Grzegorz Sarwas, Andrzej Dzieliński

**Affiliations:** Institute of Control and Industrial Electronics, Warsaw University of Technology, ul. Koszykowa 75, 00-662 Warsaw, Poland; szymon.motloch.stud@pw.edu.pl (S.M.); grzegorz.sarwas@pw.edu.pl (G.S.)

**Keywords:** fractional calculus, image processing, image fusion

## Abstract

In this paper, an analysis of the method that uses a fractional order calculus to multispectral images fusion is presented. We analyze some correct basic definitions of the fractional order derivatives that are used in the image processing context. Several methods of determining fractional derivatives of digital images are tested, and the influence of fractional order change on the quality of fusion is presented. Results achieved are compared with the results obtained for methods where the integer order derivatives were used.

## 1. Introduction

**Figure 1:**
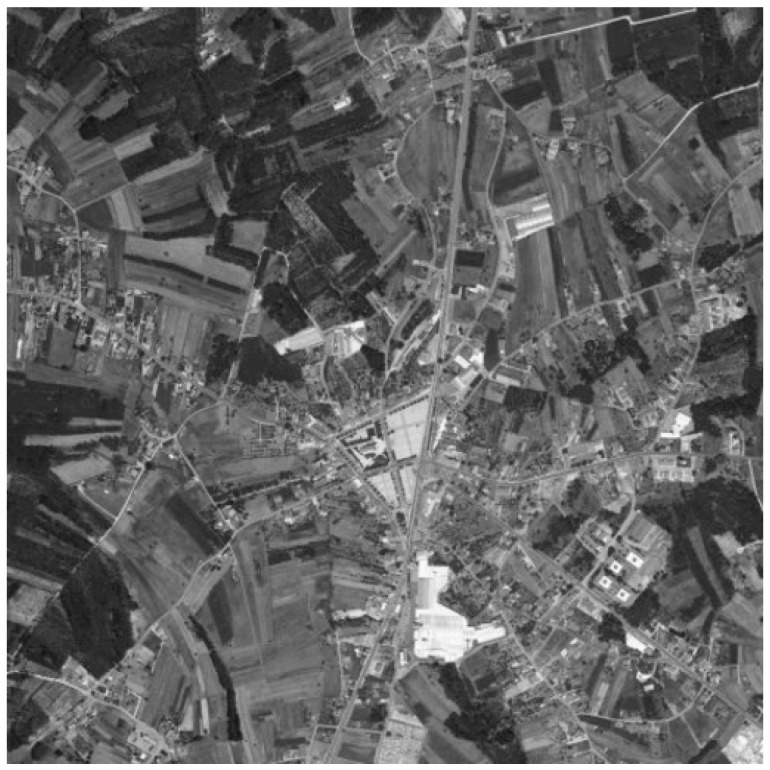
Panchromatic grayscale image. Grayscale version of Figure 3.

**Figure 2:**
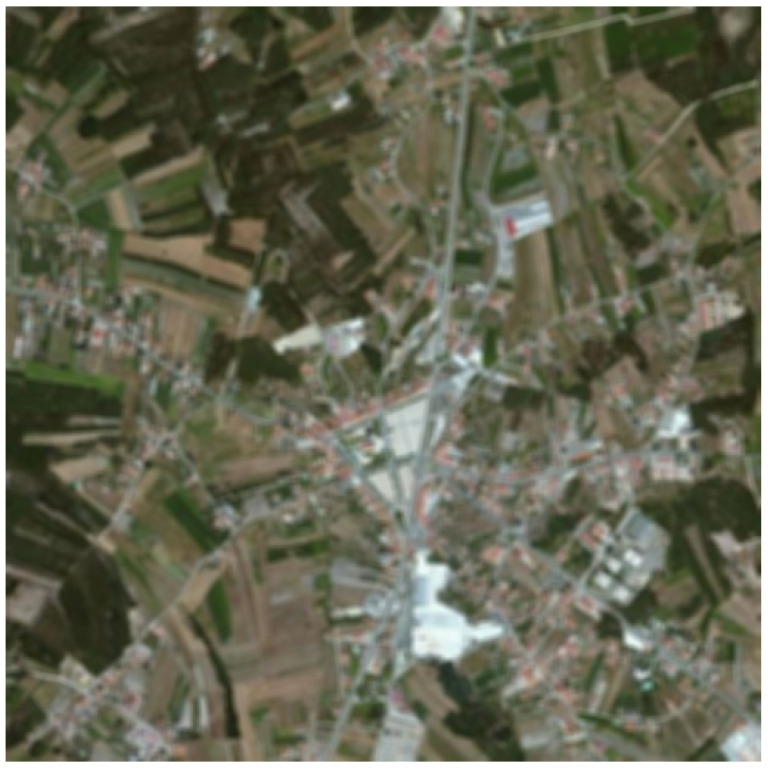
Color multispectral image. Blurred version of Figure 3.

**Figure 3:**
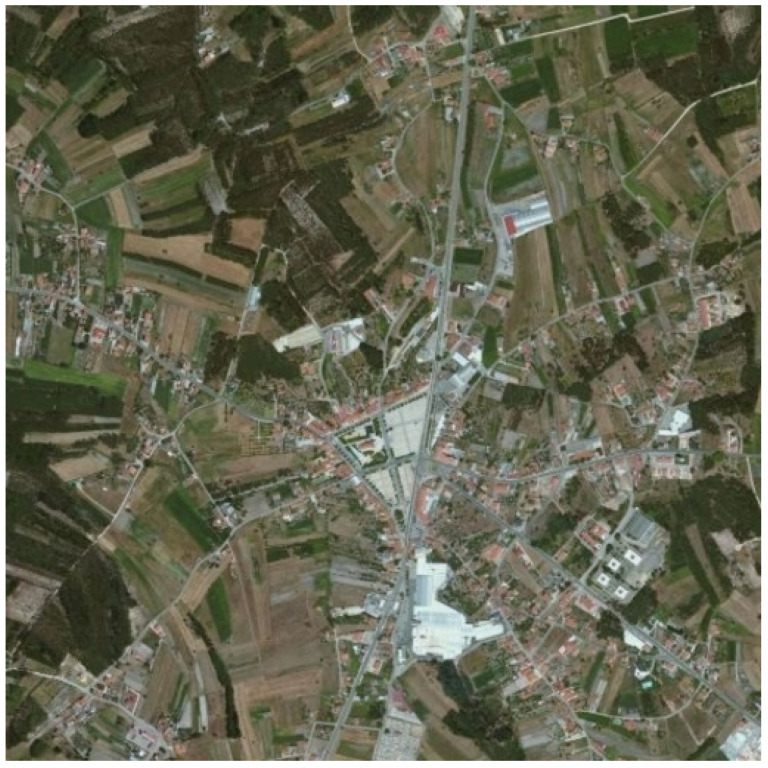
Example of panchromatic and multispectral satellite image fusion on an image from MASATI dataset v2 [3] which is available for the scientific community on demand at http://www.iuii.ua.es/datasets/masati (accessed on 8 November 2021).

In the last few decades, we can observe significant development of the image fusion theory. The main task of image fusion is to combine relevant data from different source images to generate a single image that contains richer information [1]. The most important part of this process is the effective extraction of image features and the use of appropriate fusion principles, which allow extracting useful information from source images and integrating it into the fused image without introducing any artifacts [2]. For example, fusing a panchromatic (grayscale) photo presented in Figure 1 and a multispectral (color) photo (Figure 2) is used when images show the same view. If the panchromatic image shows more details than the multispectral one, it is worth combining them, getting one color photo of good quality (more details will be visible). Figure 3 shows the effect of this kind of operation.

The presented example is only one of the various types of problems. Among them, we can highlight methods for sparse representation, multi-scale transformation, subspace, variation, neural network, saliency detection, and mixed models [4]. A combination of useful information from source images is very beneficial for subsequent applications and is widely used in such fields as photography visualization [5,6,7,8], object tracking [9,10], medical diagnosis [11,12], and remote sensing monitoring [13,14].

Recently, among image fusion methods, the emergence of algorithms based on fractional differential calculus can be observed [15,16,17,18,19]. They are applied in such problems as fingerprint detection [20], exposing of essential elements in medical images [21], brain photo analysis [22], elimination of noise in images (improved image quality) [23], or contrast enhancement [24]. As the fractional derivative started to be used relatively recently, new applications are still being found.

The adaptation of the fractional calculus to image processing problems forced researchers to develop discrete 2D approximations of fractional derivatives operators. In the literature, we can find many different definitions, both for continuous functions and their discrete approximations. However, some discrete approximations were not correctly derived. In effect, the operators that only imitate, but are not actually, the fractional operators were proposed. The literature also shows deficiencies in the analysis of individual approximations, thus answers to the questions of which of them should be used, for which problem, and which order of the derivative should be applied are essential.

In this paper, we present an analysis of the method that uses a fractional order differential to multispectral images fusion [15]. We analyze the correct basic definitions of the fractional order derivative. Original and corrected versions of the masks used in [21] have been proposed. On the experimental side, various methods of determining derivatives of digital images are tested for different datasets than the one used in [15], and the influence of fractional order change on the quality of fusion is presented. Additionally, achieved results are compared with the results obtained for methods where the integer order derivatives have been used. This novel comparison shows pros and cons of the application of fractional calculus in the context of image fusion as stated in Section 5, and motivates further studies.

## 2. Fractional Order Derivative

The non-integer (fractional) order derivative is a generalization of the traditional integer derivative of the *n*-th order (n∈N∪0, where N is a set of natural numbers) to the real or even complex order. This is a topic of mathematical analysis that can be solved in different ways. Fractional order calculus is quite a rapidly developing field nowadays, finding applications in more and more new areas.

Although this calculus was discovered over three centuries ago, it remained only a purely mathematical notion that had few or no applications for a long time. However, in the second half of the 20th century and at the beginning of the 21st century, it began to be recognized that it could be used to solve real problems. New applications for these tools were found, and more and more different models of fractional order derivatives began to emerge.

Today, fractional calculus plays an essential role in control theory, viscoelasticity, heating processes, heat conduction, biotechnology, and particle physics. The application of this calculus in image processing is also an issue that has been intensively researched in recent years. The use of some models has shown that satisfactory results have been obtained, for example, in medical image processing [21] or photo-based fingerprint recognition [20]. This calculus is used in problems where there is a memory effect since having memory is a feature of fractional order operators [21,25].

One of the challenges with the non-integer derivatives remains their physical interpretation. Contrary to integer order, the physical meaning of fractional or even real order is not entirely clear.

Another problem is that fractional order derivatives lack local character. The result of the derivative of a function depends on the entire function. This causes a significant increase in the number of necessary calculations compared to the integer order derivatives. This results in the increase of time needed for determining this type of operator.

Numerical algorithms designed to derive non-integer order derivatives contain critical, nested loops, and their complexity increases with the increasing iteration number. Programming languages such as Matlab and Python are very often used in image processing. However, these are interpreted languages and are not efficient at handling nested loops.

There are methods to speed up the calculations. One of them is the “short memory principle” [26]. It allows for getting an approximate result. Its use makes it possible not to consider the distant values of the function when calculating the derivative at a given point. At the expense of speeding up the operation, the accuracy of the final result is reduced.

Another method that can speed up the computation of non-integer derivatives is Oustaloup’s [27] method. This method allows for obtaining a correct result in a predefined frequency band. The precision of the approximation is also limited to a narrow frequency spectrum. If this band is extended, the uncertainty of the obtained result increases.

Contrary to integer order derivatives, there is no single definition for non-integer order. Many definitions have been developed, but the most frequently used are three of them: Riemann–Liouville, Caputo, and Grünwald–Letnikov. Each of these definitions has advantages and disadvantages that will be discussed. For a broad class of functions, the definitions of Riemann–Liouville and Grünwald–Letnikov are equivalent. This makes it possible to use the Riemann–Liouville derivative definition at the beginning when defining the problem and then apply the Grünwald–Letnikov definition to obtain a solution.

### 2.1. Grünwald–Letnikov Definition

This definition was proposed in 1867 [28]. For order α>0, the fractional derivative looks as follows [29]:

(1) $${}^{GL}D_{0,t}^{\alpha }f\left(t\right)=\lim_{\Delta t\to 0}\frac{1}{\Delta t^{\alpha }}\sum_{k=0}^{N}\omega_{k}^{\alpha }f\left(t-k\Delta t\right),$$

where: $\omega_{k}^{\alpha }={\left(-1\right)}^{k}\left(\begin{array}{c} \alpha \\ k \end{array}\right)$, and NΔt=t.

However, definition (1) based on the limit is practically useful only for a finite-difference implementation, hence the definition below is used:

(2) $${}^{GL}D_{0,t}^{\alpha }f\left(t\right)=\sum_{k=0}^{n-1}\frac{f^{\left(k\right)}\left(0\right)t^{-a+k}}{\Gamma (k-\alpha +1)}+\frac{1}{n-\alpha }\int_{0}^{t}{\left(t-\tau \right)}^{n-\alpha -1}f^{\left(n\right)}\left(\tau \right)d\tau ,$$

where: $n-1⩽\alpha <n\in Z^{+}$, α>0.

This definition is often used to determine the exact fractional derivative of a function. The achieved result is later used to determine the uncertainty obtained in calculating the derivative of the discrete approximation. If f(t) is sufficiently smooth, $f\left(t\right)\in C^{n}\left[0,t\right]$, then the Grünwald–Letnikov derivative is equivalent to the Riemann–Liouville definition.

In the literature, we can find GL definition in the following form:

(3) $${}^{GL}D^{\alpha }f\left(t\right)=\lim_{h\to 0}\frac{1}{h^{\alpha }}\sum_{n=0}^{\frac{t}{h}}{\left(-1\right)}^{n}\frac{\Gamma (\alpha +1)}{n!\Gamma (\alpha -n+1)}f\left(t-nh\right),$$

where: $n-1⩽\alpha <n\in Z^{+},$ α>0, Γ(x) is a gamma function.

### 2.2. Riemann–Liouville Definition

The definition of the Riemann–Liouville derivative for a real order greater than 0 of the function f(t) takes the form [30]:

(4) $${}^{RL}D_{0,t}^{\alpha }f\left(t\right)=\frac{1}{\Gamma (n-\alpha )}\frac{d^{n}}{dt^{n}}\int_{0}^{t}{\left(t-\tau \right)}^{n-\alpha -1}f\left(\tau \right)d\tau ,$$

where: $n-1<\alpha ⩽n\in Z^{+},$ α>0, Γ(x) is a gamma function.

The advantage of this definition is that the function under study does not have to be continuous at the origin. It does not have to be differentiable either [31].

### 2.3. Caputo Definition

This definition was introduced by Michele Caputo in 1967 [32]. Unlike the derivative calculated from the Riemann–Liouville definition, in this case, we do not need to define the fractional initial conditions. For a real order α∈R, Caputo’s definition of the non-integer order derivative is as follows [33]:

(5) $${}^{C}D^{\alpha }f\left(t\right)=\frac{1}{\Gamma (n-\alpha )}\int_{0}^{t}{\left(t-\tau \right)}^{n-\alpha -1}f^{\left(n\right)}\left(\tau \right)d\tau ,$$

where: t>0, n−1<α≤n, $n\in Z^{+},$ Γ(x) is a gamma function.

The value of the derivative of the non-integer order based on Caputo’s definition satisfies the important relationship:

(6) $${}^{C}D^{\alpha }A=0,$$

where *A* is a constant value. The great advantage of this method is that it includes integer initial and final conditions.

When modeling non-integer order derivatives systems, it is possible to use more than one definition. A combination of the definitions of Riemann–Liouville and Grünwald–Letnikov is often used. In addition to the mentioned definitions, many others have also been created. However, they are not used that often. De Oliveira et al. collected and described many different definitions for non-integer order derivatives [34].

### 2.4. Derivatives in Image Processing

The derivative determined on the images allows for studying the changes in brightness or color in the image. The natural application of this operator is edge detection. In this case, we need to find the derivative in two orthogonal (perpendicular) directions. They do not necessarily have to be vertical and horizontal. For example, it is possible to calculate diagonal derivatives of an image. The calculation of the first derivative of an image belongs to the group of gradient methods. They are a collection of the simplest edge detection operations to extract edges and remove the rest of the image.

The magnitude of the gradient is proportional to the rate of increase of the image function value for a given point. It also indicates how expressive the edge is. The higher the value, the more visible it is. If we want to define all the edges in the image, we should assume some limit value. For pixels whose magnitude is greater than or equal to this value, we will consider them as an edge.

Images are not continuous functions, and it is impossible to change the argument *t* by an infinitely small amount. Thus, they must be considered as discrete functions, and it is necessary to use an approximate version of an operator that will be able to act on a discrete function. The minimum value we can move in the image is one pixel. Thus, the formula for the derivative of an image takes the form:

(7) $$f^{\prime }\left(t\right)\approx \frac{f(t+1)-f(t)}{1}=f\left(t+1\right)-f\left(t\right).$$

Based on this relationship, it is possible to create many different methods of determining the gradient.

#### 2.4.1. Integer Order Derivative Mask

Many methods for determining the integer order derivatives in the images have been proposed. One such operator is Sobel’s mask [35]. It is characterized by the fact that, during averaging, weights are used, giving the analyzed point the highest value. Sobel’s mask has the following form:

(8) $$\nabla_{x}=\left[\begin{array}{ccc} 1 & 0 & -1\\ 2 & 0 & -2\\ 1 & 0 & -1 \end{array}\right],\;\nabla_{y}=\left[\begin{array}{ccc} 1 & 2 & 1\\ 0 & 0 & 0\\ -1 & -2 & -1 \end{array}\right].$$

The second order derivative can be approximated by using Laplace mask:

(9) $$\nabla^{2}=\left[\begin{array}{ccc} 0 & 1 & 0\\ 1 & -4 & 1\\ 0 & 1 & 0 \end{array}\right].$$

Based on the selected definitions of calculating fractional order derivatives, various methods are proposed that can be used in image processing. Some of them make it possible to build an appropriate mask approximating the integer order of the derivative. Other methods transform images directly. Unfortunately, many of the proposed models do not designate non-integer derivatives, despite being described in this way.

#### 2.4.2. Mask Based on Riemann–Liouville Definition

Amoako-Yirenkyi et al. [21] proposed a mask based on the generalization of Riemann–Liouville definition and for any order α∈[0,∞). However, apparently their derivation contains a mistake. In this paper, we present a correct derivation based on a standard Riemann–Liouville definition presented in Section 2.2, and for order α∈[0,1).

For an analytical function f(t), such that t∈R and α∈Q, a derivative operator is defined as:

(10) $$D_{t}^{\alpha }f\left(t\right)=\frac{1}{\Gamma (1-\alpha )}\frac{d}{dt}\int_{0}^{t}\frac{f(\tau )}{{\left(t-\tau \right)}^{\alpha }}d\tau .$$

By focusing on the integral expression in (10), we can write:

(11) $$\int_{0}^{t}\frac{f(\tau )}{{\left(t-\tau \right)}^{\alpha }}d\tau =\int_{0}^{t}f\left(\tau \right){\left(t-\tau \right)}^{-\alpha }d\tau =t^{-\alpha }*f\left(t\right)$$

and

(12) $$D_{t}^{\alpha }f\left(t\right)=\frac{1}{\Gamma (1-\alpha )}\frac{d}{dt}t^{-\alpha }*f\left(t\right).$$

This equation is for a one-dimensional function, but because an image has two dimensions, we have to transform this formula into a two-dimensional form by putting $t\to \sqrt{x^{2}+y^{2}}$. Finally, determining the directional derivatives with respect to *x* and *y*, we get the formulae for the gradient mask elements by finding the derivative in the horizontal and vertical directions, as follows:

(13) $$\begin{array}{cc} & \Theta_{x}\left(x_{i},y_{i}\right)=-\frac{\alpha \cdot x_{i}}{\Gamma (1-\alpha )}{\left(x_{i}^{2}+y_{i}^{2}\right)}^{-\alpha /2-1}, \end{array}$$

(14) $$\begin{array}{cc} & \Theta_{y}\left(x_{i},y_{i}\right)=-\frac{\alpha \cdot y_{i}}{\Gamma (1-\alpha )}{\left(x_{i}^{2}+y_{i}^{2}\right)}^{-\alpha /2-1}, \end{array}$$

where −k≤i≤k and −l≤j≤l with (2k+1)×(2l+1) is the size of the mask for every k,l≥1, and α is a constant parameter.

The determined mask of the size 5×5, proposed in [21], for the horizontal direction takes the form:

(15) $$M_{x}=\left[\begin{array}{ccccc} \frac{2\alpha \sqrt{8^{\alpha }}}{8\Gamma (1-\alpha )} & \frac{\alpha \sqrt{5^{\alpha }}}{5\Gamma (1-\alpha )} & 0 & \frac{-\alpha \sqrt{5^{\alpha }}}{5\Gamma (1-\alpha )} & \frac{-2\alpha \sqrt{8^{\alpha }}}{8\Gamma (1-\alpha )}\\ \frac{2\alpha \sqrt{5^{\alpha }}}{5\Gamma (1-\alpha )} & \frac{\alpha \sqrt{2^{\alpha }}}{2\Gamma (1-\alpha )} & 0 & \frac{-\alpha \sqrt{2^{\alpha }}}{2\Gamma (1-\alpha )} & \frac{-2\alpha \sqrt{5^{\alpha }}}{5\Gamma (1-\alpha )}\\ \frac{2\alpha \sqrt{4^{\alpha }}}{4\Gamma (1-\alpha )} & \frac{\alpha }{\Gamma (1-\alpha )} & 0 & \frac{-\alpha }{\Gamma (1-\alpha )} & \frac{-2\alpha \sqrt{4^{\alpha }}}{4\Gamma (1-\alpha )}\\ \frac{2\alpha \sqrt{5^{\alpha }}}{5\Gamma (1-\alpha )} & \frac{\alpha \sqrt{2^{\alpha }}}{2\Gamma (1-\alpha )} & 0 & \frac{-\alpha \sqrt{2^{\alpha }}}{2\Gamma (1-\alpha )} & \frac{-2\alpha \sqrt{5^{\alpha }}}{5\Gamma (1-\alpha )}\\ \frac{2\alpha \sqrt{8^{\alpha }}}{8\Gamma (1-\alpha )} & \frac{\alpha \sqrt{5^{\alpha }}}{5\Gamma (1-\alpha )} & 0 & \frac{-\alpha \sqrt{5^{\alpha }}}{5\Gamma (1-\alpha )} & \frac{-2\alpha \sqrt{8^{\alpha }}}{8\Gamma (1-\alpha )} \end{array}\right],$$

while for the vertical direction:

(16) $$M_{y}=\left[\begin{array}{ccccc} \frac{2\alpha \sqrt{8^{\alpha }}}{8\Gamma (1-\alpha )} & \frac{2\alpha \sqrt{5^{\alpha }}}{5\Gamma (1-\alpha )} & \frac{2\alpha \sqrt{4^{\alpha }}}{4\Gamma (1-\alpha )} & \frac{2\alpha \sqrt{5^{\alpha }}}{5\Gamma (1-\alpha )} & \frac{2\alpha \sqrt{8^{\alpha }}}{8\Gamma (1-\alpha )}\\ \frac{\alpha \sqrt{5^{\alpha }}}{5\Gamma (1-\alpha )} & \frac{\alpha \sqrt{2^{\alpha }}}{2\Gamma (1-\alpha )} & \frac{\alpha }{\Gamma (1-\alpha )} & \frac{\alpha \sqrt{2^{\alpha }}}{2\Gamma (1-\alpha )} & \frac{\alpha \sqrt{5^{\alpha }}}{5\Gamma (1-\alpha )}\\ 0 & 0 & 0 & 0 & 0\\ \frac{-\alpha \sqrt{5^{\alpha }}}{5\Gamma (1-\alpha )} & \frac{-\alpha \sqrt{2^{\alpha }}}{2\Gamma (1-\alpha )} & \frac{-\alpha }{\Gamma (1-\alpha )} & \frac{-\alpha \sqrt{2^{\alpha }}}{2\Gamma (1-\alpha )} & \frac{-\alpha \sqrt{5^{\alpha }}}{5\Gamma (1-\alpha )}\\ \frac{-2\alpha \sqrt{8^{\alpha }}}{8\Gamma (1-\alpha )} & \frac{-2\alpha \sqrt{5^{\alpha }}}{5\Gamma (1-\alpha )} & \frac{-2\alpha \sqrt{4^{\alpha }}}{4\Gamma (1-\alpha )} & \frac{-2\alpha \sqrt{5^{\alpha }}}{5\Gamma (1-\alpha )} & \frac{-2\alpha \sqrt{8^{\alpha }}}{8\Gamma (1-\alpha )} \end{array}\right].$$

Analyzing the masks Equations (15) and (16), it can be seen that the proposed masks do not follow Equations (13) and (14). Such inconsistency causes the final mask formulae to be contradictory to the reasoning coming from the definition of Riemann–Liouville fractional derivatives described in the formula (4). The proper masks of the size 5×5 should have the following forms:

(17) $$M_{x}=\left[\begin{array}{ccccc} \frac{2\alpha \sqrt{8^{\alpha }}}{8^{\alpha +1}\Gamma \left(1-\alpha \right)} & \frac{\alpha \sqrt{5^{\alpha }}}{5^{\alpha +1}\Gamma \left(1-\alpha \right)} & 0 & \frac{-\alpha \sqrt{5^{\alpha }}}{5^{\alpha +1}\Gamma \left(1-\alpha \right)} & \frac{-2\alpha \sqrt{8^{\alpha }}}{8^{\alpha +1}\Gamma \left(1-\alpha \right)}\\ \frac{2\alpha \sqrt{5^{\alpha }}}{5^{\alpha +1}\Gamma \left(1-\alpha \right)} & \frac{\alpha \sqrt{2^{\alpha }}}{2^{\alpha +1}\Gamma \left(1-\alpha \right)} & 0 & \frac{-\alpha \sqrt{2^{\alpha }}}{2^{\alpha +1}\Gamma \left(1-\alpha \right)} & \frac{-2\alpha \sqrt{5^{\alpha }}}{5^{\alpha +1}\Gamma \left(1-\alpha \right)}\\ \frac{2\alpha \sqrt{4^{\alpha }}}{4^{\alpha +1}\Gamma \left(1-\alpha \right)} & \frac{\alpha }{\Gamma (1-\alpha )} & 0 & \frac{-\alpha }{\Gamma (1-\alpha )} & \frac{-2\alpha \sqrt{4^{\alpha }}}{4^{\alpha +1}\Gamma \left(1-\alpha \right)}\\ \frac{2\alpha \sqrt{5^{\alpha }}}{5^{\alpha +1}\Gamma \left(1-\alpha \right)} & \frac{\alpha \sqrt{2^{\alpha }}}{2^{\alpha +1}\Gamma \left(1-\alpha \right)} & 0 & \frac{-\alpha \sqrt{2^{\alpha }}}{2^{\alpha +1}\Gamma \left(1-\alpha \right)} & \frac{-2\alpha \sqrt{5^{\alpha }}}{5^{\alpha +1}\Gamma \left(1-\alpha \right)}\\ \frac{2\alpha \sqrt{8^{\alpha }}}{8^{\alpha +1}\Gamma \left(1-\alpha \right)} & \frac{\alpha \sqrt{5^{\alpha }}}{5^{\alpha +1}\Gamma \left(1-\alpha \right)} & 0 & \frac{-\alpha \sqrt{5^{\alpha }}}{5^{\alpha +1}\Gamma \left(1-\alpha \right)} & \frac{-2\alpha \sqrt{8^{\alpha }}}{8^{\alpha +1}\Gamma \left(1-\alpha \right)} \end{array}\right],$$

while for the vertical direction:

(18) $$M_{y}=\left[\begin{array}{ccccc} \frac{2\alpha \sqrt{8^{\alpha }}}{8^{\alpha +1}\Gamma \left(1-\alpha \right)} & \frac{2\alpha \sqrt{5^{\alpha }}}{5^{\alpha +1}\Gamma \left(1-\alpha \right)} & \frac{2\alpha \sqrt{4^{\alpha }}}{4^{\alpha +1}\Gamma \left(1-\alpha \right)} & \frac{2\alpha \sqrt{5^{\alpha }}}{5^{\alpha +1}\Gamma \left(1-\alpha \right)} & \frac{2\alpha \sqrt{8^{\alpha }}}{8^{\alpha +1}\Gamma \left(1-\alpha \right)}\\ \frac{\alpha \sqrt{5^{\alpha }}}{5^{\alpha +1}\Gamma \left(1-\alpha \right)} & \frac{\alpha \sqrt{2^{\alpha }}}{2^{\alpha +1}\Gamma \left(1-\alpha \right)} & \frac{\alpha }{\Gamma (1-\alpha )} & \frac{\alpha \sqrt{2^{\alpha }}}{2^{\alpha +1}\Gamma \left(1-\alpha \right)} & \frac{\alpha \sqrt{5^{\alpha }}}{5^{\alpha +1}\Gamma \left(1-\alpha \right)}\\ 0 & 0 & 0 & 0 & 0\\ \frac{-\alpha \sqrt{5^{\alpha }}}{5^{\alpha +1}\Gamma \left(1-\alpha \right)} & \frac{-\alpha \sqrt{2^{\alpha }}}{2^{\alpha +1}\Gamma \left(1-\alpha \right)} & \frac{-\alpha }{\Gamma (1-\alpha )} & \frac{-\alpha \sqrt{2^{\alpha }}}{2^{\alpha +1}\Gamma \left(1-\alpha \right)} & \frac{-\alpha \sqrt{5^{\alpha }}}{5^{\alpha +1}\Gamma \left(1-\alpha \right)}\\ \frac{-2\alpha \sqrt{8^{\alpha }}}{8^{\alpha +1}\Gamma \left(1-\alpha \right)} & \frac{-2\alpha \sqrt{5^{\alpha }}}{5^{\alpha +1}\Gamma \left(1-\alpha \right)} & \frac{-2\alpha \sqrt{4^{\alpha }}}{4^{\alpha +1}\Gamma \left(1-\alpha \right)} & \frac{-2\alpha \sqrt{5^{\alpha }}}{5^{\alpha +1}\Gamma \left(1-\alpha \right)} & \frac{-2\alpha \sqrt{8^{\alpha }}}{8^{\alpha +1}\Gamma \left(1-\alpha \right)} \end{array}\right].$$

We compare the results obtained for correctly determining masks in the conducted experiments. Still, we also analyze the results obtained for the incorrect masks to assess the impact of this error on the results achieved.

#### 2.4.3. Eight-Directions Non-Integer Order Mask

In [15], a method was described that allows for building an eight-direction mask approximating non-integer order derivatives. This model is based on the Grünwald–Letnikov definition presented in (3).

For images, the minimum value of *h* change is equal to one pixel. After differentiating the finite function f(x) with respect to *x*, based on the expression (3), one can get the Formulae (19) and (20):

(19) $$\frac{\partial^{\alpha }f\left(x,y\right)}{\partial x^{\alpha }}≅f\left(x,y\right)+\left(-\alpha \right)f\left(x-1,y\right)+\frac{\left(-\alpha )(-\alpha +1\right)}{2!}f\left(x-2,y\right),$$

(20) $$\frac{\partial^{\alpha }f\left(x,y\right)}{\partial y^{\alpha }}≅f\left(x,y\right)+\left(-\alpha \right)f\left(x,y-1\right)+\frac{\left(-\alpha )(-\alpha +1\right)}{2!}f\left(x,y-2\right).$$

Based on the above expressions, it is possible to build masks determining approximations of non-integer derivatives in eight different directions. Following [15], the final mask composed of all eight directional masks should be more robust to image rotation due to its symmetry. The mask components for eight directions appear as follows:

Derivative mask in direction $x_{+}$: $\left[\begin{array}{ccc} 0 & 1 & 0\\ 0 & -\alpha & 0\\ 0 & \frac{\alpha^{2}-\alpha }{2} & 0 \end{array}\right].$
Derivative mask in direction $x_{-}$: $\left[\begin{array}{ccc} 0 & \frac{\alpha^{2}-\alpha }{2} & 0\\ 0 & -\alpha & 0\\ 0 & 1 & 0 \end{array}\right].$
Derivative mask in direction $y_{+}$: $\left[\begin{array}{ccc} 0 & 0 & 0\\ 1 & -\alpha & \frac{\alpha^{2}-\alpha }{2}\\ 0 & 0 & 0 \end{array}\right].$
Derivative mask in direction $y_{-}:$ $\left[\begin{array}{ccc} 0 & 0 & 0\\ \frac{\alpha^{2}-\alpha }{2} & -\alpha & 1\\ 0 & 0 & 0 \end{array}\right].$
Derivative mask in direction left upper diagonal: $\left[\begin{array}{ccc} \frac{\alpha^{2}-\alpha }{2} & 0 & 0\\ 0 & -\alpha & 0\\ 0 & 0 & 1 \end{array}\right].$
Derivative mask in direction left lower diagonal: $\left[\begin{array}{ccc} 0 & 0 & 1\\ 0 & -\alpha & 0\\ \frac{\alpha^{2}-\alpha }{2} & 0 & 0 \end{array}\right].$
Derivative mask in direction right upper diagonal: $\left[\begin{array}{ccc} 0 & 0 & \frac{\alpha^{2}-\alpha }{2}\\ 0 & -\alpha & 0\\ 1 & 0 & 0 \end{array}\right].$
Derivative mask in direction right lower diagonal: $\left[\begin{array}{ccc} 1 & 0 & 0\\ 0 & -\alpha & 0\\ 0 & 0 & \frac{\alpha^{2}-\alpha }{2} \end{array}\right].$
Based on the presented mask components, the resultant eight-direction mask can be obtained: $M=\left[\begin{array}{ccccc} \frac{\alpha^{2}-\alpha }{2} & 0 & \frac{\alpha^{2}-\alpha }{2} & 0 & \frac{\alpha^{2}-\alpha }{2}\\ 0 & -\alpha & -\alpha & -\alpha & 0\\ \frac{\alpha^{2}-\alpha }{2} & -\alpha & 8 & -\alpha & \frac{\alpha^{2}-\alpha }{2}\\ 0 & -\alpha & -\alpha & -\alpha & 0\\ \frac{\alpha^{2}-\alpha }{2} & 0 & \frac{\alpha^{2}-\alpha }{2} & 0 & \frac{\alpha^{2}-\alpha }{2} \end{array}\right],$ where α is a derivative order.

This mask appears to approximate fractional derivatives and will be further applied to the image fusion study. Its use is presented in [15] and may be a good reference point for other methods.

#### 2.4.4. FFT Approximation of a Fractional Order Derivative

In [36], a method for determining the fractional order derivative was shown. It is based on the Riemann–Liouville definition of the following form:

(21) $${}^{RL}D_{a,t}^{\alpha }f\left(t\right)=\frac{1}{\Gamma (n-\alpha )}\frac{d^{n}}{dt^{n}}\int_{a}^{t}{\left(t-\tau \right)}^{n-\alpha -1}f\left(\tau \right)d\tau ,$$

where α∈R is a fractional order of differ-integral of the function f(t) and, for n∈N∪0, we have: n−1<α≤*n* for α>0, n=0 for α≤0.

The Fourier transform of the Riemann–Liouville fractional derivatives with the lower bound a=−∞ is equal to:

(22) $$F\left(D^{\alpha }f\left(x\right)\right)={\left(j\omega \right)}^{\alpha }F\left(\omega \right).$$

For any two-dimensional function g(x,y) absolutely integrable in (−∞,∞)×(−∞,∞), the corresponding 2D Fourier transform is as follows [30]:

(23) $$G\left(\omega_{1},\omega_{2}\right)=\int g\left(x,y\right)e^{-j(\omega_{1}x+\omega_{2}y)}dxdy.$$

Therefore, we can write the formulae for fractional order derivatives as:

(24) $$\begin{array}{c} D_{x}^{\alpha }g=F^{-1}\left({\left(j\omega_{1}\right)}^{\alpha }G\left(\omega_{1},\omega_{2}\right)\right),\\ D_{y}^{\alpha }g=F^{-1}\left({\left(j\omega_{2}\right)}^{\alpha }G\left(\omega_{1},\omega_{2}\right)\right), \end{array}$$

where $F^{-1}$ is the inverse 2D continuous Fourier transform operator.

Finally, the result of image fractional derivative will be determined by the real part of the sum of the inverse transforms:

(25) $$D^{\alpha }g=ℜ\left(D_{x}^{\alpha }g+D_{y}^{\alpha }g\right),$$

where *ℜ* is a real part of a complex function.

## 3. Methods and Metrics Used

For our experiments, we used the component substitution method proposed in [37]. The basis of this image fusion method is to extract the details of images by determining the difference between a panchromatic image and a linear combination of low-quality multispectral image channels. This method is effective when two combined images contain almost the same information.

The image fusion algorithm without a fractional order derivative was proposed in [38] and can be written as follows:

(26) $$M_{k}^{H}=M_{k}^{L}+g_{k}\left(P-I\right),$$

where

(27) $$I=\sum_{i=1}^{N}\omega_{i}M_{i}^{L},$$

and $M_{k}^{H}$ is the *k*-th band of the fused image, $M_{k}^{L}$ is *k*-th band of the low-resolution multispectral image, $\omega_{i}$ represents the band weight, and $g_{k}$ is a constant gain determined according to the relationship from [39]:

(28) $$g_{k}=\frac{cov(M_{k}^{L},I)}{var(I)},$$

for k=1,2,…,N, P is a panchromatic image, I is a linear combination of a low-resolution multispectral image, *N* indicates the number of bands covering the spectral signature of the panchromatic image, cov(X,Y) denotes covariance between the *X* and *Y* images, and var(X) is a variance of image X. The band coefficients are determined in accordance with the AIHS approach (Adaptive Intensity-Hue-Saturation) [40], which achieves good results in this issue.

Azarang et al. in [15] proposed a modification of this method. Using the non-integer order derivative of the difference (P–I) should strengthen the edges and improve the fusion results. The modified form of the algorithm (26) can be written as follows:

(29) $$M_{k}^{H}=M_{k}^{L}+g_{k}\times m*\left(P-I\right),$$

where *m* is a proposed mask and ∗ is a convolution operator.

### Metrics

A problem with comparing the quality of image fusion results is the lack of an appropriate metric. Our experiments described in Section 4 support the claim that commonly used metrics such as the ones defined below are not always able to clearly determine which approach results in better visual quality of an image. Because of this, in the experiments, a selection of metrics is used.

To evaluate results, the following metrics were applied:

- Root Mean Square Error (RMSE)—measures the changes in pixel values of the input band of the multispectral image *R* and the sharpened image *F*. This metric is fundamental if the images contain large, uniform areas. This error is calculated using the following formula [41]:
  (30) $$RMSE=\sqrt{\frac{1}{m\times n}\sum_{i=1}^{m}\sum_{j=1}^{n}{\left|R\left(i,j\right)-F\left(i,j\right)\right|}^{2}}.$$
  Ideal value of this error is 0.
- Relative dimensionless global error in synthesis (ERGAS) is a global quality factor. This error is sensitive to the change in the average pixel value of the image and the dynamically changing range. Its value tells about the amount of spectral distortion in the image. It is expressed as [41]:
  (31) $$ERGAS=100\cdot \frac{h}{l}\sqrt{\frac{1}{N}\sum_{i=1}^{N}{\left(\frac{RMSE(i)}{\mu (i)}\right)}^{2}},$$
  where: $\frac{h}{l}$ is the ratio of the number of pixels of a panchromatic image to the number of pixels of a multispectral image, μ(i) is the mean of i-th band, while *N* is the total number of bands. In the case of this metric, we aim for the error rate to be close to zero.
- Spectral angle mapper (SAM) calculates spectral similarities by finding a spectral angle between two spectral vectors with a common origin. The length of a spectrum vector $L_{p}$ is calculated by the following equation:
  (32) $$L_{p}=\sqrt{\sum_{\lambda =1}^{M}\rho_{\lambda }^{2}},$$
  and the spectral angle θ is calculated as [42]:
  (33) $$\theta =\cos^{-1}\left(\frac{\sum_{\lambda =1}^{M}\rho_{\lambda }\rho_{\lambda }^{{}^{\prime }}}{L_{\rho }L_{\rho^{\prime }}}\right),$$
  where: $L_{\rho }$ is the length of the endmember vector, $L_{\rho^{\prime }}$ is the length of the modeled spectrum vector, $\rho_{\lambda }$—reflection of the endmember. In the case of this metric, we aim for the error rate to be close to zero.
- Correlation coefficient (CC)—shows the spectral correlation between two images. The value of this coefficient for the sharpened image *F* and the input multispectral image *R* is determined as follows [41]:
  (34) $$\sum CC\left(R,F\right)=\frac{\sum_{mn}^{}\left(R_{mn}-\bar{R}\right)\left(F_{mn}-\bar{F}\right)}{\sqrt{\sum_{mn}^{}{\left(R_{mn}-\bar{R}\right)}^{2}\left(\sum_{mn}^{}{\left(F_{mn}-\bar{F}\right)}^{2}\right)}}.$$
  $\bar{R}$ and $\bar{F}$ mean the average values of the *F* and *R* images, while *m* and *n* denote the shape of the images. The optimal value for this coefficient is 1.
- Universal Image Quality Index (UIQI) is defined as [41]:
  (35) $$Q\left(R,F\right)≜\frac{\sigma_{RF}}{\sigma_{R}\cdot \sigma_{F}}\times \frac{2\cdot \bar{R}\cdot \bar{F}}{{\bar{R}}^{2}+{\bar{F}}^{2}}\times \frac{2\cdot \sigma_{R}\cdot \sigma_{F}}{\sigma_{R}^{2}+\sigma_{F}^{2}}.$$
  The first factor presents the correlation coefficient between the images *R* and *F*, while the second factor is the luminance distance, and the last represents the contrast distortion. $\sigma_{RF}$ denotes the covariance between the images *R* and *F*. $\bar{R}$ and $\bar{F}$ are the mean values, $\sigma_{R}^{2}$ and $\sigma_{F}^{2}$ denote the standard deviation values of the *R* and *F* images, respectively. The best value for this index is 1. It can be reached if *R* equals *F* for all pixels.
- Relative Average Spectral Error (RASE) is computed using the RMSE value using the following relation [41]:
  (36) $$RASE=\frac{100}{\mu }\sqrt{\frac{1}{N}\sum_{i=1}^{N}RMSE^{2}\left(B_{i}\right)},$$
  where μ is the mean radiance of the *N* spectral bands and $B_{i}$ represents the i-th band of the input multispectral image. An optimal value of this error is equal to 0.

## 4. Experiments

For the experiments, a set of photos in PNG format, taken by the satellite from the Bing maps [3], has been used. The creator of this collection divided the data into categories such as land and water. Our experiments were focused only on a part with land-based images containing 1078 images. This collection includes good-quality panchromatic photos. Based on this collection, two image sets were prepared. The first was the result of saving color photos in grayscale, representing high-quality panchromatic photos. The second contained multispectral color photos with reduced quality due to the use of a blurring mask.

As the first experiment, the mean error values for the image fusion algorithm for the basic version, without additional derivatives [43], were checked. Its description and an indication of where the examined modification can be applied can be found in Section 3. The result of the experiment is shown in Table 1. Then, for the prepared data set, the method proposed in [15], based on a Grünwald–Letnikov definition that allows for building an eight-direction mask defined in Section 2.4.2, approximating the non-integer order derivatives, was examined. The results achieved for this method are presented in Table 2. The experiments with masks presented in Section 2.4.2 were shown in Table 3 and the improved version of this mask in Table 4. An experiment was also carried out in which the fractional derivative was determined using the Fast Fourier transform described in Section 2.4.4. The results of this experiment are presented in Table 5. For comparison, the experiments with the first derivative using Sobel’s filter, and the second derivative using Laplace operator, the description of which can be found in the Section 2.4.1, were added. The results of Sobel’s and Laplace’s filters for image fusion are presented in Table 6.

The results presented in the tables show that the best result of fusion for 4 out of 6 metrics was obtained by using an eight-direction mask proposed in [15] for derivative order α=0.1 (see Table 2). This result is slightly better than results obtained without fractional order derivatives (see Table 1). It can be observed that increasing the fractional order of derivative from 0.1 to 0.9 worsens the results. However, for the order in the range <0.6; 0.9>, this mask achieved the worst results from all examined methods.

The best results of all the proposed modifications were obtained for the FFT method and the order of α=0.2, which can be seen in Table 5. Using the FFT-based derivative, the smallest fusion errors were obtained for the order α=0.2. Up to the order of 0.4, the error increases slightly, while, from value 0.5, the errors increase with the order, but much slower than with the original method for which errors above 0.5 are already significant.

**Figure 4:**
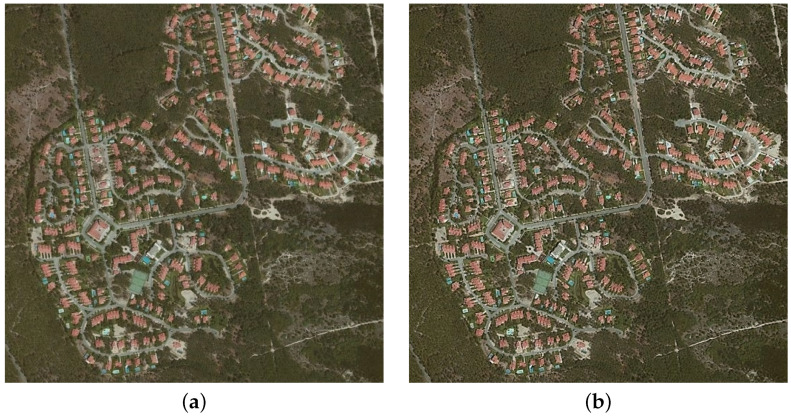
Comparison of the image fusion of the original algorithm based on an eight-direction approximation mask with an FFT solution. (**a**) Eight-direction mask for derivative order α=0.1 with metrics: ERGAS = 0.31914, SAM = 0.17813, RASE = 1.2257, RMSE = 0.99474, UIQI = 0.14063, CC = 0.14238. (**b**) FFT solution for derivative order α=0.2 with metrics: ERGAS = 0.60386, SAM = 0.32631, RASE = 2.322, RMSE = 1.8845, UIQI = 0.13603, CC = 0.1355.

Although the lowest error results obtained by the FFT-based algorithm is twice as high as the best results obtained with the eight-directional mask, the visual assessment of the final images calls into question the determined error values. The FFT fusion images look optically similar, just as precise, and sometimes appear sharper, which can be observed in Figure 4. It confirms that, in image processing, better metric values do not always guarantee higher visual quality.

An incorrect mask proposed by [21] achieved the worst results from tested fractional order operators (see Table 3). The corrected version of this mask improved results a little bit (see Table 4). For these two masks, we can observe that maximum errors were obtained around the order of 0.5.

The Sobel and Laplace filters to approximate the first and the second order derivative gave abysmal results for all metrics (see Table 6).

## 5. Conclusions

In the paper, the improved, corrected versions of the edge detecting masks based on fractional derivative have been proposed. The masks were implemented with different approximations of this derivative. Analyzed modification of different approximations did not improve the quality of image fusion much. In some cases, the introduced changes while improving the quality metrics of the fused images worsened their visual quality for the tested set.

Analysis of achieved metrics’ values shows that the original fractional order solution achieved the best metrics for order α=0.1; however, the fusion error for orders higher than 0.6 increased dramatically. More stable results were achieved by using the FFT algorithm for computing fractional order derivatives. Despite the error of the FFT algorithm for α=0.2 being two times higher, visual verification indicates that this algorithm received sharper fused images. This confirms the thesis that there are currently no metrics that would unambiguously assess the quality of image fusion.

The integer order derivative method produces a result that completely disrupts the algorithm. Comparing the results with various methods for determining non-integer order derivatives shows that these methods often have a much greater chance of success in applications where standard methods for integer orders do not bring positive results. Edge reinforcement is essential in this application. Fractional derivatives accomplish this to varying degrees, creating a greater chance that a particular combination will be better suited for a given application.

The experiments presented show the potential of applying fractional derivatives in the image fusion context. However, there is still a lot of future research required. It would be worth testing the operation of the best-performing filter for different data sets. In addition, new masks based on different definitions of a fractional derivative may lead to better results. A well-known problem of image quality metrics not clearly reflecting the image’s visual quality should be addressed in future work.

## Figures and Tables

**Table 1:** Results obtained without derivative.

ERGAS	SAM	RASE	RMSE	UIQI	CC
1.7451	1.1342	6.7379	5.909	0.98136	0.99716

**Table 2:** Results of the original method of determining fractional derivatives proposed in [15].

Order	Metrics					
	ERGAS	SAM	RASE	RMSE	UIQI	CC
0.1	1.6453	1.1691	6.3661	5.5738	0.98422	0.99564
0.2	1.7475	1.2770	6.7936	5.9460	0.98373	0.98983
0.3	2.1839	1.5340	8.5277	7.4752	0.97679	0.97738
0.4	3.0660	2.1112	11.997	10.538	0.95787	0.95496
0.5	4.5489	3.3488	17.805	15.660	0.91720	0.91805
0.6	6.9785	5.8739	27.301	24.028	0.83875	0.86105
0.7	11.228	10.824	43.894	38.644	0.70026	0.77827
0.8	19.989	20.423	78.087	68.756	0.48409	0.66629
0.9	46.807	39.958	182.76	160.92	0.21384	0.52810

**Table 3:** Results for computing derivative using an incorrect mask proposed in [21].

Order	Metrics					
	ERGAS	SAM	RASE	RMSE	UIQI	CC
0.1	4.5152	1.3413	17.530	15.473	0.88896	0.88148
0.2	5.1704	1.5590	20.094	17.747	0.86107	0.84455
0.3	5.9243	1.8679	23.043	20.363	0.82702	0.80213
0.4	6.5676	2.2037	25.559	22.594	0.79688	0.76689
0.5	6.9541	2.4420	27.070	23.934	0.77856	0.74642
0.6	6.9822	2.4629	27.180	24.033	0.77734	0.74512
0.7	6.5961	2.2276	25.670	22.695	0.79588	0.76581
0.8	5.8131	1.8263	22.609	19.980	0.83259	0.80885
0.9	4.8188	1.4427	18.719	16.529	0.87657	0.86469

**Table 4:** Results for computing derivative using a corrected mask.

Order	Metrics					
	ERGAS	SAM	RASE	RMSE	UIQI	CC
0.1	4.4760	1.3296	17.377	15.338	0.89117	0.88362
0.2	4.9592	1.4868	19.269	17.015	0.87140	0.85687
0.3	5.4032	1.6495	21.006	18.555	0.85230	0.83232
0.4	5.6730	1.7611	22.061	19.490	0.84035	0.81750
0.5	5.7194	1.7810	22.243	19.651	0.83825	0.81492
0.6	5.5455	1.7059	21.563	19.047	0.84595	0.82434
0.7	5.1971	1.5694	20.200	17.839	0.86116	0.84346
0.8	4.7644	1.4203	18.506	16.338	0.87941	0.86745
0.9	4.3864	1.3010	17.027	15.026	0.89466	0.88845

**Table 5:** Results for derivative based on FFT.

Order	Metrics					
	ERGAS	SAM	RASE	RMSE	UIQI	CC
0.1	3.2273	2.1970	12.622	11.105	0.95322	0.94970
0.2	3.1091	2.0855	12.154	10.690	0.95696	0.95382
0.3	3.1971	2.0222	12.486	10.984	0.95455	0.95083
0.4	3.4573	1.9987	13.489	11.871	0.94665	0.94145
0.5	3.8598	2.0138	15.047	13.250	0.93311	0.92554
0.6	4.3800	2.0704	17.065	15.034	0.91338	0.90253
0.7	4.9985	2.1740	19.469	17.157	0.88683	0.87174
0.8	5.6996	2.3289	22.196	19.564	0.85300	0.83274
0.9	6.4703	2.5401	25.197	22.210	0.81182	0.78561

**Table 6:** Results for integer order masks.

Mask	ERGAS	SAM	RASE	RMSE	UIQI	CC
Sobel	23.220	20.218	90.583	80.032	0.26922	0.32230
Laplacian	12.742	5.7724	49.64	43.723	0.41921	0.35566

## Data Availability

Not applicable.
